# Lifestyle Interventions Targeting Body Weight Changes during the Menopause Transition: A Systematic Review

**DOI:** 10.1155/2014/824310

**Published:** 2014-05-26

**Authors:** Janet Jull, Dawn Stacey, Sarah Beach, Alex Dumas, Irene Strychar, Lee-Anne Ufholz, Stephanie Prince, Joseph Abdulnour, Denis Prud'homme

**Affiliations:** ^1^Faculty of Graduate Studies, Institute of Population Health, University of Ottawa, 1 Stewart Street, Room 300, Ottawa, ON, Canada K1N 6N5; ^2^School of Nursing, Faculty of Health Sciences, University of Ottawa, 451 Smyth Road, Room RGN 1118, Ottawa, ON, Canada K1H 8M5; ^3^Centre for Practice Changing Research, Clinical Epidemiology Program, Ottawa Hospital Research Institute, 501 Smyth Road, Box 201B, Ottawa, ON, Canada K1H 8L6; ^4^School of Human Kinetics, Faculty of Health Sciences, University of Ottawa, 125 University Private, MNT 366, Ottawa, ON, Canada K1N 6N5; ^5^Département de Nutrition, Université de Montréal et CRCHUM, 2405 Côte Ste-Catherine, Pavillon Liliane-de-Stewart, Montréal, QC, Canada H3T 1A1; ^6^Health Sciences Library, University of Ottawa, 451 Smyth Road, RGN Health Sciences Library, Ottawa, ON, Canada K1H 8M5; ^7^Prevention and Rehabilitation Division, University of Ottawa Heart Institute, 40 Ruskin Street, Ottawa, ON, Canada K1Y 4W7; ^8^Institut de Recherche de l'Hôpital Montfort, 745 Building A, Suite 202, Montreal Road, Ottawa, ON, Canada K1K 0T1

## Abstract

*Objective.* To determine the effectiveness of exercise and/or nutrition interventions and to address body weight changes during the menopause transition. *Methods.* A systematic review of the literature was conducted using electronic databases, grey literature, and hand searching. Two independent researchers screened for studies using experimental designs to evaluate the impact of exercise and/or nutrition interventions on body weight and/or central weight gain performed during the menopausal transition. Studies were quality appraised using Cochrane risk of bias. Included studies were analyzed descriptively. *Results.* Of 3,564 unique citations screened, 3 studies were eligible (2 randomized controlled trials, and 1 pre/post study). Study quality ranged from low to high risk of bias. One randomized controlled trial with lower risk of bias concluded that participation in an exercise program combined with dietary interventions might mitigate body adiposity increases, which is normally observed during the menopause transition. The other two studies with higher risk of bias suggested that exercise might attenuate weight loss or weight gain and change abdominal adiposity patterns. *Conclusions.* High quality studies evaluating the effectiveness of interventions targeting body weight changes in women during their menopause transition are needed. Evidence from one higher quality study indicates an effective multifaceted intervention for women to minimize changes in body adiposity.

## 1. Introduction

Twenty-four percent of Canadians are obese and a further 37% are overweight using measured height and weight data from the 2007–2009 Canadian Health Measures Survey [1]. Further, over half (58%) of the Canadian women aged 40 to 59 years are considered overweight or obese [2]. Poor eating habits and physical inactivity contribute to the increasing prevalence of overweight and obese individuals [3]. The majority of middle-aged women do not regularly participate in exercise and/or fail to report healthy nutrition practices such as consuming five or more vegetables and fruits per day [4, 5]. Evidence also suggests a positive trend with aging and weight gain [1]. These trends mark the need to identify lifestyle interventions that address these healthy behaviours and their contribution to addressing midlife weight gain in women, to minimize the risks for obesity and related cardiometabolic complications.

In their midlife, women undergo the biologic transition into menopause. This menopause transition is divided into several stages and clinically has been categorized by international criteria developed to help assess women's reproductive stage [6]. The early menopause transition stage is specifically defined by menstrual cycles varying by seven or more days from regular cycles; the next stage is defined by intervals of skipped cycles or amenorrhea of at least 60 days. The third stage begins following the first year without menstruation and is defined as the end of perimenopause; which is then followed by a stage of early postmenopause and lasting up to six years. During the transition, women can experience vasomotor symptoms such as hot flashes and/or night sweats which may continue for five years or more years after the last menstrual period [7]. These symptoms typically occur in women ages 40 to 65 years [8] and are more prevalent in obese women [9, 10]. Other changes that occur during the menopause transition are changes in body composition and increase in abdominal fat mass as well as associated alterations in cardiometabolic risks due to hormone-related decreases in energy expenditure and fat oxidation [7, 11]. Health conditions (or problems) associated with central obesity in women include coronary heart disease, hypertension, type 2 diabetes, cancer, osteoarthritis, and gall bladder disease [12].

Lifestyle interventions to minimize gains in fat mass and changes in body composition and body fat distribution predominantly include exercise and healthy nutrition [13, 14]. Current guidelines recommend (a) assessing factors contributing to overweight (body mass index (BMI) 25–29.9 kg·m^2^) and obesity (BMI ≥ 30 kg·m^2^) in adults and (b) intervening with counseling and treatment of obesity [15]. Specific interventions include encouraging individuals to set realistic lifestyle goals, referral to weight loss programs, pharmacotherapy, or surgical interventions. Despite these guidelines, it is unclear what effect lifestyle interventions, such as exercise and/or healthy nutrition, have on weight gain, body composition, and/or body fat distribution in women, if performed specifically during the menopause transition stage. While systematic reviews of the literature evaluating interventions to prevent weight gain exist for more broadly defined populations, there are limited studies evaluating interventions for women during this critical midlife menopausal transition stage. Furthermore, the sequel of the menopause transition stage are often including increases in fat mass and abdominal adiposity and the associated risks of developing cardiometabolic complications are greater compared to the other periods in a women's life, when there are also significant hormone changes (e.g., menarche and pregnancy) [16].

Ultimately, this review was conducted to provide a synthesis of current evidence and identify gaps in the knowledge around effective exercise and nutrition interventions that address body weight and body composition changes in women specifically during the menopause transition stage. To date, only one known literature review on this topic exists and it was not systematic [17]. Further, it focused on the effects of hormonal changes on body weight and body composition during the transition to menopause [17].

The objective of this study was to determine the effectiveness of exercise and/or nutrition interventions on changes in body weight, body composition, and body fat distribution in women specifically during the menopause transition stage.

## 2. Methods

A systematic review was conducted using a protocol developed a priori according to the Cochrane Handbook for Systematic Reviews of Interventions [21] and the Preferred Reporting Items for Systematic Reviews and Meta-Analyses (PRISMA) statement [22]. Systematic reviews apply explicit methods to conduct an extensive search, select appropriate research studies, critically appraise the studies, and synthesize studies to answer specific questions [22, 23].

### 2.1. Comprehensive Search Strategy

The search strategy was developed collaboratively with an academic reference librarian (LAU) and researchers investigating the effect of menopause transition on body weight regulation (Table 1). Population, intervention, comparator, and outcome (PICO) [24] were used for developing the search strategy and focused on key words related to menopause transition, physical activity/exercise, and nutrition interventions (see Table 2). The search strategy was designed for Medline and adapted for EMBASE, CINAHL, AMED, PsycINFO, Global Health, and SPORTDiscus. Grey literature and hand searching were conducted by using included articles to look for related articles in PubMed and checking reference lists of included articles. During the screening process for inclusion/exclusion of studies, if the published information was unclear, authors were contacted by email with requests for additional details on their study.

### 2.2. Selection Process

Included studies evaluated exercise, or nutrition interventions targeting specifically women in the menopause transition stage and evaluated impacts on body weight and/or central and abdominal weight gain. The menopause transition is divided into several stages and clinically has been categorized by international criteria developed to help assess women's reproductive stage [6]. All intervention designs were included: randomized controlled trials (RCT's), crossover designs, case controlled studies, and pre-/post tests. Screening and selection of each of the included studies was conducted independently by two reviewers (JJ, SP; JJ, SB) using standardized forms and included three phases: (I) title screen; (II) abstract screen; and (III) full article screen, with screening decisions recorded.

For the level (I) title screen, the citation titles identified by the search strategy were reviewed to identify any studies about women and body weight and/or abdominal obesity during the menopause transition stage. Titles identified as “include” or “unsure” by any reviewer were retained for second level screening. For level (II) screening, abstracts were evaluated using the PICO inclusion/exclusion criteria (Table 2). Level (III) screening involved a full text review of each article using the standardized screening form. Consensus on differences was reached in a discussion with a third reviewer (DS).

### 2.3. Data Extraction and Analysis

Data extraction was conducted independently by two reviewers (JJ, SB) and using standardized forms. Collected data included characteristics of the intervention, characteristics of the study design, characteristics of the participants, and findings. The menopausal status of study participants was described based on the international STRAW+10 staging system's stages of reproductive aging in women [6]. The Cochrane Collaboration Risk of Bias Tool was used to appraise study quality for the RCT's [21, 25] and the Critical Appraisal Skills Programme (CASP) checklist was used for other study designs [26]. Differences in extracted data or appraisal ratings between the reviewers were resolved through discussion with consensus reached and reasons for decisions documented.

Given the heterogeneity of included study designs and few studies included, a descriptive analysis was conducted.

## 3. Results

**Figure 1:**
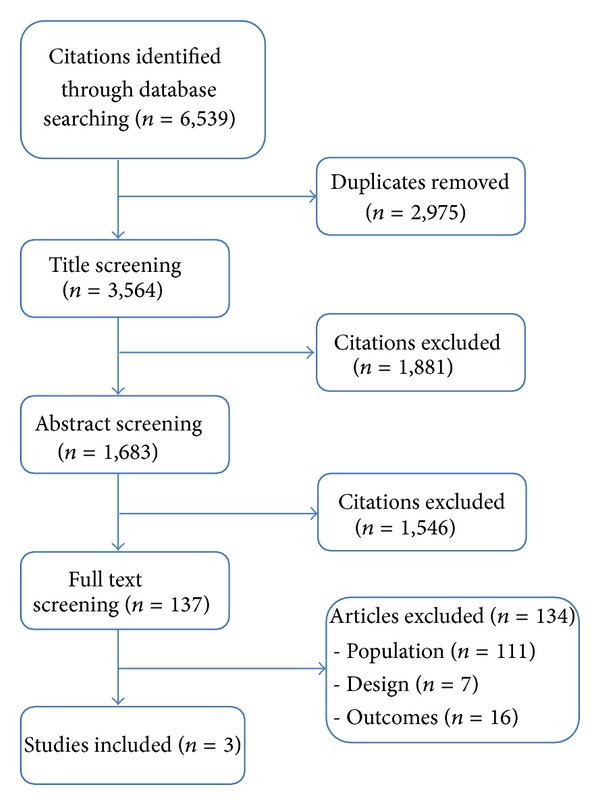
Flow diagram for screening process.

Of the 3,564 unique citations identified, only 3 studies were eligible for inclusion (Figure 1). Of the 137 citations in full text review, 134 were excluded: 111 included women whose menopausal status was not explicitly defined as being specifically in the stage of menopause transition (e.g., menopausal status undefined, mixed pre-/peri-/postmenopausal), 7 were not experimental designs (e.g., editorials or summaries discussing evidence on the role of exercise, diet, and diet and exercise), and 16 examined other outcomes (e.g., bone health and quality of life) (in the appendix).

### 3.1. Characteristics of Included Studies

Included were two RCTs [18, 19] and one pre-/post study [20] published between 2003 and 2011 (Table 3). The number of participants ranged from 24 [19] to 535 [18]. The loss of participants due to study withdrawal or lost to follow-up was described in all three included studies [18–20]. Additionally, participants self-identified their menopausal status in all three studies. Participants from the five-year RCT ranged in age from 44 to 50 years at the start of enrollment for the five-year study [18]. The other two studies included women in varying menopausal stages; one reported baseline information for perimenopausal and postmenopausal women with ages ranging from 40 to 59 years of age, although the intervention and results were reported as separate perimenopausal and postmenopausal groups of women [19]. The other study identified women during the menopause transition (last menstruation ≥ 60 and ≤365 days) separately from premenopausal (last menstruation < 60 days) or postmenopausal women (last menstruation > 365 days) and resulted in a wider age range [20]. Quality appraisals of the RCTs identified one study with low risk of bias [18]. The other RCT provided insufficient detail to judge risk of bias for allocation concealment, blinding, and selective reporting of outcomes [19]. The pre-/post study had lower risk of bias but did not report adequate detail to judge risk of bias related to confounding factors, follow-up, and results [20].

### 3.2. Characteristics of Interventions

Study interventions were exercise with diet or exercise only (Table 4). One study evaluated highly supported (i.e., coaching, education) caloric and dietary restrictions combined with exercise over 20 weeks, followed by ongoing and less intensive dietary and exercise interventions for another 48 months [18]. Another study evaluated a 12-week exercise intervention with participants randomly assigned to a circuit training program or control group [19]. The third study evaluated endurance exercise using a Nordic walking program over 12 weeks [20].

In the studies that utilized exercise as an intervention, participants were either not instructed in their dietary habits [19] or were asked to refrain from increasing their daily fat intake and to avoid changing their nutrition routines [20]. The other study focused on supporting significant changes to both exercise and dietary lifestyle habits [18].

### 3.3. Characteristics of Outcome Measures

All three studies used different measures of adiposity and body fat distribution. One study focused predominantly on changes in BMI and waist-to-hip ratio [19]. The other studies measured overall gains and/or losses in body weight reported as BMI with percentage body fat [18] or total kilograms of fat [20]. Abdominal obesity was measured using waist circumference [18, 20] or waist-to-hip ratio only [19].

#### 3.3.1. Combined Exercise and Dietary Intervention, RCT (*n* = 1)

Compared to controls, participants randomized to an exercise and dietary intervention at 54 months were more likely to be at or below baseline weight (55% versus 26%) and experienced a decreased BMI and greater loss of percentage of body fat for all three follow-up measures, including decreased waist circumference (*P* < 0.001) [18]. While both intervention and control groups were found to have decreased waist circumference measurements at follow-up, the greater changes were measured in the intervention group [18]. The mean changes in body weight were −0.2 lbs. (−0.1 kg, SD = 5.2 kg) in the intervention group and +5.2 lbs. (2.4 kg, SD = 4.9 kg) in the control group.

#### 3.3.2. Exercise Only Intervention Only, RCT (*n* = 1)

Participants randomized to circuit training significantly decreased the mean values of their waist-to-hip ratio (*P* < 0.05) between baseline and week 12, whereas those in the control group saw no significant changes over the 12-week follow-up period. Significant changes in BMI were not observed in either group (*P* > 0.05) [19] and attributed to the minimal changes in body weight for both groups during the 12 weeks of the study. Overall, participants randomized to a 12-week circuit training exercise program only decreased their waist-to-hip ratio.

#### 3.3.3. Exercise Only Intervention, Pre-/Post study (*n* = 1)

Compared to baseline, participants exposed to moderate endurance exercise over 12 weeks saw reductions in BMI, total body fat, and waist circumference (*P* ≤ 0.05) [20].

## 4. Discussion

### 4.1. Summary of Main Results

This systematic review was focused on determining the effectiveness of exercise and/or nutrition interventions on mitigating changes in body weight, body composition, and body fat distribution in women specifically in menopause transition stage. Despite extensive searching of published and unpublished research, only three relevant studies were identified. Most studies were excluded due to poorly defining the menopausal status of study populations, lacking an intervention study, or lacking assessment outcomes related to body weight or body composition. The included studies described various study environments, participant characteristics, and interventions.

Of the three included studies, one higher quality trial with minimal risk of bias showed that compared to usual activities in the control group, women exposed to a program of combined exercise and caloric restriction dietary interventions for 54 weeks had improved body weight and reduced abdominal adiposity [18]. As well, significant reductions in waist circumference and body fat were maintained beyond four years. Despite using a less rigorous study design and having a shorter follow-up period, another study concluded that a Nordic walking program might reduce weight gain during the menopause transition stage [20]. Similarly, a study implementing a circuit training program found that the waist-to-hip ratio could be decreased with exercise, even while measures of BMI were not significantly influenced [19]. While limited in number, these studies suggest that exercise or both exercise and caloric reduction interventions may be able to disrupt the process and patterns of weight gain and change in body fat distribution during the menopause transition stage.

These findings are consistent with guidelines on the prevention and management of obesity that recommend lifestyle intervention as the first approach for preventing or treating obesity [27]. More specifically, lifestyle recommendations include regular exercise and a reduced-energy diet. These recommendations are supported by other studies addressing excess weight and obesity in middle-aged women and obese adults [28, 29]. The paucity of studies that have investigated the effects of lifestyle intervention on weight, body fat, and body fat distribution specifically in women in the menopausal transition stage indicates that better designed studies are needed to support an evidence-based approach to managing and preventing women's body weight changes during this specific important life stage.

### 4.2. Periodic Clinical Measurements as an Intervention

Of the three included studies, only one study [18] suggested the potential impact of periodic clinical measurements as an intervention to attenuate weight gain during the menopause transition stage. Clinical measurements included weight, waist circumference, blood pressure, lipids, glucose, and level of physical activity and nutrient intake by questionnaire. Of note is that 26% of controls were at or below baseline weight at the 54-month visit and the group showed a significant decrease in waist circumference without being exposed to the intensive lifestyle intervention. Therefore, it is possible that the women in the control group may have been influenced by the periodic clinical measurements at 6, 18, 30, 42, and 54 months [18] and this is consistent with another study [30]. A longitudinal observational study attempting to document the effect of menopause transition in healthy nonobese premenopausal women found no significant weight gain after 5-year follow-up. In this study, there was no structured intervention other than that of yearly clinical measures of body weight, waist circumference, body composition, blood pressure, lipids profile, glucose, physical activity, and a food intake journal [30].

### 4.3. Reproductive Aging Is Poorly Defined in Studies

Selection of participants using self-reported menopausal status was described for all three studies and details on characteristics of participants were limited [18–20]. Participants included in the studies varied in age from 40 to 62 years and reproductive aging was understood as distinct from generalized physical aging. Of concern was the fact that many of the excluded studies examining weight gain in women from pre- to postmenopause did not describe menopausal status and failed to clearly define the reproductive stage of participants.

Reporting of the data in ways that obscure potential variability between women of different reproductive ages is an issue and has been previously identified as contributing to the development of less effective interventions for managing menopausal symptoms [31]. Criteria defining the stages of menopause can better situate studies examining effects of reproductive aging and help women and health professionals to effectively address issues, such as identification of critical stages for increased risk of adiposity. For this systematic review, the menopause transition stage was described based on the international STRAW+10 staging system's stages of reproductive aging in women [6]. Future studies could benefit from standard reporting of menopausal status using these internationally approved criteria.

Several factors have been shown to be influential during women's experience of menopause. While the prevalence of excess weight and obesity is high within the general population; population subgroups such as those with lower socioeconomic status have been identified as being at particular risk [32]. The findings of this systematic review indicate that a narrow segment of the population has been studied (i.e., predominantly white, middle-class women). Other key factors in reproductive aging (e.g., race, ethnicity, culture, geography, and socioeconomic status) are not prominent in the study of body weight changes during the menopause transition [31], despite longitudinal evidence that socioeconomic status is related to BMI, weight gain, and certain weight control practices within nationally representative samples of women [33]. One included study in this review identified this gap in the literature addressing diversity in research and reporting on women and changes in adiposity associated with menopause [19] and cited this as a rationale for their study. Indeed, the attrition rate for participants in the study was attributed to the socioeconomic structures limiting participation of women in their active lifestyles [19]. Hence, the findings of this systematic review highlight the need to ensure that consistent definitions are applied in defining reproductive aging stage, and that the characteristics of subpopulations of women are included as an integral part of research on interventions that target body weight, body composition, and body fat distribution during the menopause transition stage.

### 4.4. Limitations and Strengths

The potential limitations of this review included a lack of a standardized definition for the population of women in the menopause transition within the literature as described above; poor indexing of studies in databases; and the limited time frame (<26 weeks) over which two of the three studies were conducted. Given the poor indexing of studies in electronic databases, it is possible that some studies were missed; however, there is transparency in the extensive search strategy used. Sustained weight loss, up to and longer than one year, is considered a standard in studies of management of overweight and obesity [28]. However, only one of the three included studies was conducted for longer than one year with the other two studies being only 12-week duration. During study screening, sensitivity was favoured over specificity, resulting in larger numbers of studies screened and ultimately excluded. As well, the studies relied on self-reports by women on their menopausal status. Strengths of this review included the comprehensive search strategy which was developed in collaboration with an academic librarian, use of two independent reviewers at each screening stage, and the iterative and ongoing consultation with an interprofessional team of researchers having expertise in obesity, reproductive aging, and systematic review methodology.

## 5. Conclusions

Few studies have measured the effect of exercise and/or dietary interventions on women's body weight or body composition specifically during the menopause transition stage. Evidence from one large higher quality RCT indicates that women should exercise and eat a caloric restricted diet during the menopause transition stage to prevent weight gain and abdominal fat gain. The review also identified the need for use of common terminology for defining reproductive age of study participants as described by the STRAW+10 staging system [6]. Common terminology would facilitate synthesizing results across studies. Finally, further research should focus on more rigorous study methods using durations of one year or longer. Ideally, studies should be sensitive to the potential relationships between cultural norms, socioeconomic status, and body weight changes during the menopause transition stage. Subsequent results would be more likely to help women and their health professionals choose effective interventions for addressing changes in body weight and achieving a body composition defined by women and their care professionals as healthy.

## Figures and Tables

**Table 1:** Medline Search Strategy.

	Database: Ovid MEDLINE(R) In-Process and Other Non-Indexed Citations and Ovid MEDLINE(R) <1948 to Present>
	Search Strategy:
(1)	menopause/or perimenopause/or premenopause/(26010)
(2)	“perimenopaus*”.ab,ti. (2638)
(3)	“peri-menopaus*”.ab,ti. (216)
(4)	“premenopaus*”.ab,ti. (12032)
(5)	“pre-menopaus*”.ab,ti. (1186)
(6)	climacteric.ab,ti. (3389)
(7)	menopaus$.ab,ti. (29513)
(8)	1 or 2 or 3 or 4 or 5 or 6 or 7 (51438)
(9)	Exercise/(52421)
(10)	Physical Fitness/(18078)
(11)	Exercise Therapy/(20391)
(12)	exp Sports/(88445)
(13)	Motor Activity/(60911)
(14)	exercis$.ab,ti. (160690)
(15)	yoga.ab,ti. (1081)
(16)	(aerobics or “physical therapy” or “physical activity” or “physical inactivity”).ab,ti. (46541)
(17)	(fitness adj3 (class$ or regime$ or program$)).ab,ti. (883)
(18)	(walking or running or jogging or swimming or cycling or bicycling).ab,ti. (100485)
(19)	(“tai chi” or “tai ji”).ab,ti. (526)
(20)	“physical training”.ab,ti. (3882)
(21)	danc$.ab,ti. (3061)
(22)	sedentary behavio?r.ab,ti. (582)
(23)	(physical adj3 (inactivity or activity or training)).ab,ti. (43594)
(24)	(“weight lifting” or “strength training” or “resistance training” or “circuit weight training”).ab,ti. (4695)
(25)	exertion.ab,ti. (8780)
(26)	sports.ab,ti. (21251)
(27)	“lifestyle intervention*”.ab,ti. (1583)
(28)	(“cognitive behavio*ral” and “weight loss”).ab,ti. (144)
(29)	exp Diet Therapy/(35073)
(30)	Nutrition Therapy/(556)
(31)	(diets or diet or dieting).ab,ti. (194636)
(32)	(diet$ adj (modif$ or therapy or intervention$ or strateg$)).ab,ti. (7935)
(33)	(low calorie or “calorie control$” or “healthy eating”).ab,ti. (3368)
(34)	(nutrition$ adj2 (modif$ or therapy or intervention$ or strateg$)).ab,ti. (5810)
(35)	or/9–32 (617979)
(36)	body weight changes/or weight gain/(17952)
(37)	Overweight/(5768)
(38)	body mass index/or skinfold thickness/(58989)
(39)	Waist Circumference/(1525)
(40)	“body weight”.ab,ti. (119849)
(41)	“fat distribution”.ab,ti. (3555)
(42)	“body composition”.ab,ti. (16098)
(43)	“waist circumference”.ab,ti. (7570)
(44)	“body mass index”.ab,ti. (66808)
(45)	“skin fold”.ab,ti. (1130)
(46)	“abdominal fat”.ab,ti. (3072)
(47)	“adipose tissue”.ab,ti. (32751)
(48)	Hot Flashes/(1699)
(49)	Vasomotor System/(8540)
(50)	(flush$ or flash$ or sweat$).ab,ti. (44468)
(51)	vasomotor.ab,ti. (9284)
(52)	or/36–51 (319221)
(53)	8 and 35 and 52 (1536)
(54)	limit 53 to yr = “1865–2010” (1523)
(55)	limit 54 to (english or french) (1457)
(56)	from 55 keep 1–1457 (1457)

**Table 2:** Criteria for study eligibility.

Criteria	Included	Excluded
Population	Perimenopausal women and early postmenopausal women during the transition to menopause and up to 6 years postmenopause (typical ages from 40 to 65) and BMI range from 20 to 40 kg·m^2^	Premenopausal women (e.g., no menstrual irregularities) or more than 6 years postmenopausal

Intervention	Interventions including nutrition and/or physical activity (e.g., aerobic exercise such as running and biking) as the primary component	Intervention did not involve nutrition and/or physical activity as the primary component Excluded supplements, such as vitamins

Comparator	Any comparator.	

Outcomes	Body weight changes (e.g., weight changes, prevention of gain, and maintenance) and/or abdominal obesity	Primary outcome reports on bone density

Study Design	Experimental designs (e.g., RCT, interrupted time series, and pre-/posttest)	Nonexperimental designs (cohort study)

**Table 3:** Characteristics of included trials (*N* = 3).

First author (year)	Country	Design	Participants	Menopausal status (at time of recruitment)	Comparisons	Study quality rating
Simkin-Silverman 2003 [18]	USA	RCT	535 women (intervention = 260 control = 275)	Less than 3 months amenorrhea in past 6 months	Two phased dietary and behavioural program versus control for 54 months	6/6 criteria met (low risk of bias)

Ogwumike 2011 [19]	Nigeria	RCT	24 women completed full study (intervention = 13 control = 11)	Irregular menstruation that has not ceased entirely, in the past year	Circuit training program, 3 times/week versus control for 12 weeks	3/6 criteria met (unclear risk of bias)

Hagner 2009 [20]	Poland	Pre/Post	53 women	Final menstrual period: premenopausal <60 days perimenopausal ≥60 days, ≤365 days, and postmenopausal >365 days	Nordic walking program (moderate intensity exercise) for 12 weeks	9/12 criteria met (lower risk of bias for cohort study)

RCT: randomized controlled trial.

**Table 4:** Characteristics of intervention (*N* = 3).

Element	Simkin-Silverman et al. (2003) [18]	Ogwumike et al. (2011) [19]	Hagner et al. (2009) [20]
Type	Dietary and physical activity	Physical activity	Physical activity
	Group 1: intervention Phase I:	Group 1: intervention	90-minute Nordic walking 3 times per week
	(a) Set weight loss goals	(a) 10 station circuit consisting of muscular and cardiovascular endurance, flexibility, coordination, and abdominal and pelvic floor muscle exercises	
	(b) Eat low-fat foods (1300 kcal/day)	(b) Target heart exercise rate was calculated for each woman at 60 to 80 percent of heart rate reserve	
	(c) Sessions for adherence to meal plan	(c) 3 times per week on alternate days; grouped into one of three groups in the order of their baseline cardiorespiratory fitness	
	(d) 1000–1500 kcal/week physical activity	(d) 40- to 60-minute circuit exercises plus 10 minutes warm up/cool down	
	(e) Consultations to develop and adhere to physical activity routine	Group 2: Control-assessment only	No control group
	(f) Self-monitoring and feedback on diet and physical activity	Reported at four weekly intervals for assessments (adiposity and flexibility)	
	Phase II: (a) Group meetings (b) Refresher programs (c) Increased caloric intake as weight goals met Group 2: Control-assessment only		

Duration	Phase 1 = 20 weeks	12 weeks	12 weeks
	Phase 2 = 48 months		

## Appendix

### A. Reasons for Exclusion (Total *n* = 134)

#### A.1. Menopausal Status Undefined (*n* = 59)

P. Abedi, M. H. Lee, and M. Kandiah et al., in “Diet intervention to improve cardiovascular risk factors among Iranian postmenopausal women,”* Nutrition Research and Practice*, vol. 4, no. 6, pp. 522–527, 2010, recruited “postmenopausal women” to enter the trial without additional details: “no menstruation for at least 12 months.”

E. J. Aiello, Y. Yasui, and S. S. Tworoger et al., in “Effect of a yearlong, moderate-intensity exercise intervention on the occurrence and severity of menopause symptoms in postmenopausal women,”* Menopause*, vol. 11, no. 4, pp. 382–388, 2004, recruited “postmenopausal women” ages 50–75 without additional details, postmenopausal status unknown.

S. Alhassan, S. Kim, A. Bersamin, A. C. King, and C. D. Gardner, in “Dietary adherence and weight loss success among overweight women: results from the A TO Z weight loss study,”* International journal of obesity*, vol. 32, no. 6, pp. 985–991, 2008, did not recruit women for menopausal status, not defined as postmenopausal.

D. Anderson, K. Mizzari, V. Kain, and J. Webster, in “The effects of a multimodal intervention trial to promote lifestyle factors associated with the prevention of cardiovascular disease in menopausal and postmenopausal Australian women,”* Health Care for Women International*, vol. 27, no. 3, pp. 238–253, 2006, recruited women with unknown menopausal status, ages 50 to 65 years.

B. Andersson, J. Seidell, K. Terning, and P. Bjorntorp, in “Influence of menopause on dietary treatment of obesity,”* Journal of Internal Medicine*, vol. 227, no. 3, pp. 173–181, 1990, recruited women defined as “pre- and postmenopausal,” menopausal status unclear.

S. R. Arefhosseini, C. A. Edwards, D. Malkova, and S. Higgins, in “Effect of advice to increase carbohydrate and reduce fat intake on dietary profile and plasma lipids,”* Annals of Nutrition and Metabolism*, vol. 54, no. 2, pp. 138–144, 2009, recruited “postmenopausal women” ages 56.2+/−6.5 years, last menses 3+ years before study.

H. Arguin, I. Dionne, and M. Senechal et al., in “Short- and long-term effects of continuous versus intermittent restrictive diet approaches on body composition and the metabolic profile in overweight and obese postmenopausal women: A pilot study,”* Menopause*, vo. 19, no. 8, pp. 870–876, 2012, recruited women with “no menstruation for more than 1 year” and no additional details on menopausal status.

B. J. Arsenault, M. Cote, and A. Cartier et al., in “Effect of exercise training on cardiometabolic risk markers among sedentary, but metabolically healthy overweight or obese post-menopausal women with elevated blood pressure,”* Atherosclerosis*, vol. 207, no. 2, pp. 530–533, 2009, recruited women 45–75 years, no further details on menopausal status.

E. A. Asbury, C. Slattery, A. Grant, L. Evans, M. Barbir, and P. Collins, in “Cardiac rehabilitation for the treatment of women with chest pain and normal coronary arteries,”* Menopause*, vol. 15, no. 3, pp. 454–460, 2008, recruited women 57.3 ± 8.6 years, no menopausal criteria included.

T. M. Asikainen, S. Miilunpalo, and P. Oja, et al., in “Randomised, controlled walking trials in postmenopausal women: the minimum dose to improve aerobic fitness?”* British Journal of Sports Medicine*, vol. 36, no. 3, pp. 136–194, 2002, recruited women defined as 2–10 years past the “onset of menopause.”

As per M. Aubertin, E. T. Poehlman, and I. J. Dionne, in “Effect of regular physical activity on body composition in older women: independent of the type of exercise,”* Canadian Journal of Applied Physiology*, vol. 28, no. pp. S28, 2003, the menopausal status was undefined, and participants were 50–70 years of age.

M. Aubertin-Leheudre, C, Lord, A. Khalil, and I. J. Dionne, in “Effect of 6 months of exercise and isoflavone supplementation on clinical cardiovascular risk factors in obese postmenopausal women: a randomized, double-blind study,”* Menopause*, vol. 14, no. 4, pp. 624–629, 2007, recruited “postmenopausal women” (without menses for at least the past 12 months) 50–70 years of age, no additional details.

M. Aubertin-Leheudre, C. Lord, A. Khalil, and I. J. Dionne, in “Isoflavones and clinical cardiovascular risk factors in obese postmenopausal women: A randomized double-blind placebo-controlled trial,”* Journal of Women's Health*, vol. 17, no. 8, pp. 1363–1369, 2008, recruited “postmenopausal women” defined as without menses for at least the past 12 months, 50–70 years of age.

Y. A. Baek, K. N. Kim, Y. A. Lee, and N. Chang, in “The effect of nutrition education on visceral fat reduction and diet quality in postmenopausal women,”* Korean Journal of Nutrition*, vol. 41, no. 7, pp. 634–644, 2008, recruited 101 women, undefined menopausal status.

N. D. Barnard, A. R. Scialli, G. Turner-McGrievy, A. J. Lanou, and J. Glass, in “The effects of a low-fat, plant-based dietary intervention on body weight, metabolism, and insulin sensitivity”,* American Journal of Medicine*, vol. 118, no. 9, pp. 991–997, 2005, recruited “postmenopausal women,” age range 44–73, no additional details.

J. W. Bea, E. C. Cussler, S. B. Going, R. M. Blew, L. L. Metcalfe, and T. G. Lohman, in “Resistance training predicts 6-yr body composition change in postmenopausal women,”* Medicine and Science in Sports and Exercise*, vol. 42, no. 7, pp1286–1295, 2010, recruited postmenopausal women defined as “40–65 years of age, surgical or natural menopause (3.0–10.9 years).”

V. Bonganha, M. Conceição, M. Chacon-Mikahil, and V. Madruga, in “Response of the Resting Metabolic Rate after 16 Weeks of Resistance Training in Postmenopausal Women,”* Revista Brasileira de Medicina do Esporte*, vol. 17, no. 5, pp. 350–353, 2011, recruited postmenopausal women (minimum absence of 12 months of menstruation), with no additional details.

V. Bonganha, D. Modeneze, V. Madruga, and R. Vilarta, in “Effects of resistance training (RT) on body composition, muscle strength and quality of life (QoL) in postmenopausal life,”* Archives of Gerontology* &* Geriatrics*, vol. 54, no. 2, pp. 361–365, 2012, recruited postmenopausal women (minimum absence of 12 months of menstruation), with no additional details.

S. Calzavarini, S. Volpato, P. Caruso, A. Passaro, R. Fellin, and F. Bernardi, in “Modulation of coagulation factor levels and thrombin generation parameters by a healthy diet in premenopausal middle-aged women with moderate cardiovascular risk. XXII Congress of the International Society of Thrombosis and Haemostasis 2009 Jul 11–Jul 16; Boston, MA United States,”* Journal of Thrombosis and Haemostasis*, vol. 7, no. S2, pp. 1056, 2009, recruited participants (women) with undefined menopausal status.

S. Chai, S. Hooshmand, R. L. Saadat, M. Payton, K. Brummel-Smith, and B. H. Arjmandi, in “Daily apple versus dried plum: impact on cardiovascular disease risk factors in postmenopausal women,”* Journal of the Academy of Nutrition and Dietetics*, vol. 112, no. 8, pp. 1158–1168, 2012, recruited participants who included “A total of 236 healthy postmenopausal women (1 to 10 years past menopause).”

Y. W. Chen and P. Watson, in “The effects of an individualized diet and exercise program on body fat levels in Taiwanese females aged 40–60,”* Proceedings of the Nutrition Society of New Zealand*, vol. 27, pp. 107, 2002, recruited participants (women) who were 40–60 years of age and without details on menopausal status.

F. F. Colakoglu, in “The effect of callisthenic exercise on physical fitness values of sedentary women,”* Science and Sports*, vol. 23, no. 6, pp. 306–309, 2008, gave participants (women) age comparisons (i.e., 30–44; 45–55; 55+) without detail on menopausal status.

M. M. Cowan and L. W. Gregory, in “Responses of pre- and post-menopausal females to aerobic conditioning,”* Medicine and Science in Sports and Exercise*, vol. 17, no. 1, pp. 138–143, 1985, recruited two participant groups: one group of women had not had menses for 12 months or more (postmenopausal) and the other group had regular menses (“premenopausal”), with no additional details.

D. Y. Seo, S. R. Lee, and H. K. Kim et al., in “Independent beneficial effects of aged garlic extract intake with regular exercise on cardiovascular risk in postmenopausal women”* Nutrition Research and Practice*, vol. 6, no. 3, pp. 226–231, 2012, recruited women for age, with no detail on menopausal status.

As per A. T. Darestani, F. Hosseinpanah, F. Tahbaz, Z. Amiri, R. T. Darestani, and M. Hedayati, in “Effects of conjugated linoleic acid supplementation on body composition and leptin concentration in post-menopausal women,”* Iranian Journal of Endocrinology and Metabolism*, vol. 12, no. 1, pp. 48, 2010, the menopausal status was undefined: “postmenopausal women.”

As per S. Drapeau, E. Doucet, R. Rabasa-Lhoret, M. Brochu, D. Prud'homme, and P. Imbeault, in “Improvement in insulin sensitivity by weight loss does not affect hyperinsulinemia-mediated reduction in total and high molecular weight adiponectin: a MONET study,”* Applied Physiology, Nutrition, and Metabolism*, vol. 36, no. 2, pp. 191–200, 2011, the menopausal status was undefined: “postmenopausal women.”

As per S. Faghih, A. Abadi, M. Hedayati, and M. Kimiagar, in “The effect of the combination of restricted energy diet and low-fat milk or calcium supplement on iron status of premenopausal overweight or obese women,”* Clinical Nutrition, Supplement*, vol. 7, no. 1, pp. 238-239, 2012, the menopausal status was undefined: “menopausal women.”

As per K. Foster-Schubert, C. M. Alfano, and C. R. Duggan, in “Effect of diet and exercise, alone or combined, on weight and body composition in overweight-to-obese postmenopausal women* Obesity*, vol. 20, no. 8, pp. 1628–1638, 2012, the menopausal status was undefined: “postmenopausal women aged 50 to 75 years.”

As per C. M. Friedenreich, C. G. Woolcott, and A, McTiernan et al., in “Adiposity changes after a 1-year aerobic exercise intervention among postmenopausal women: a randomized controlled trial,”* International Journal of Obesity*, vol. 35, no. 3, pp. 427–435, 2011, the menopausal status was undefined: “postmenopausal women aged 50 to 74 years.”

As per M. Friedrich, in “Effect of health-promoting education in nutrition and changes in eating habits on levels of insulin, lipids and lipoproteins in the blood of obese women being in climacterium,”* Polish Journal of Food and Nutrition Sciences*, vol. 7, no. 1, pp. 125–134, 1998, the menopausal status was undefined: “postmenopausal and 48–58 years of age.”

As per G. Garnier, S. Garnier, I. Gaubert, and G. Auneau, in “Follow up of an active walking program on sedentary and moderately obese postmenopausal women,”* Climacteric*, vol. 14, pp. 73-74, 2011, the menopausal status was undefined: “women aged 60+/−5 years”.

M. M. Gordon, M. J. Bopp, and L. Easter et al., in “Effects of dietary protein on the composition of weight loss in post-menopausal women,”* Journal of Nutrition, Health and Aging*, vol. 12, no. 8, pp. 505–509, 2008, recruited women defined as having no menses for one year+ and 50–70 years of age.

J. S. Green, P. R. Stanforth, and T. Rankinen et al., in “The effects of exercise training on abdominal visceral fat, body composition, and indicators of the metabolic syndrome in postmenopausal women with and without estrogen replacement therapy: The HERITAGE Family Study,”* Metabolism, Clinical and Experimental*, vol. 53, no. 9, pp. 1192–1196, 2004, recruited participants, women with no menses for 3 months+ and 46–59 years of age, with no further details.

M. D. Haub, T. R. Simons, C. M. Cook, V. M. Remig, E. Al-Tamimi, and C. A. Holcomb, in “Calcium-fortified beverage supplementation on body composition in postmenopausal women,”* Nutrition Journal*, vol. 4, pp. 21, 2005, recruited participants: women greater than one year with no menses, 48–72 years of age, with no further details.

M. Hohanka, M. Irwin, and S. R. Heckbert et al., in “Serum lipoproteins in overweight/obese postmenopausal women: a one-year exercise trial,”* Medicine and Science in Sports and Exercise*, vol. 38, no. 2, pp. 231–239, 2006 reported no menopausal status, women 50–75 years of age.

B. V. Howard, J. E. Manson, and M. L. Stefanick et al., in “Low-fat dietary pattern and weight change over 7 years: the women's health initiative dietary modification trial,”* Journal of the American Medical Association*, vol. 295, no. 1, pp. 39–49, 2006, reported no menopausal status, women 50–79 years of age.

A. J. Huang, L. L. Subak, and R. Wing, et al., in “Program to reduce incontinence by diet and exercise, investigators. An intensive behavioral weight loss intervention and hot flushes in women,”* Archives of Internal Medicine*, vol. 170, no. 13, pp. 1161–1167, 2010, recruited participants, women with no menses 12 months+ and hot flashes, with no further details.

M. L. Irwin, Y. Yasui, and C. M. Ulrich et al., in “Effect of exercise on total and intra-abdominal body fat in postmenopausal women: A randomized controlled trial,”* Journal of American Medical Association*, vol. 289, no. 3, pp. 323–330, 2003, recruited participants, “postmenopausal women for at least 24 months, aged 50 to 74 years.”

G. Kaddissy and N. Lattouf, in “Daily integration of the regular aquatic rhythmic activity in women's life during menopause and its role in the prevention of the metabolic syndrome,”* Kinesitherapie*, vol. 11, no. 118, pp. 48–53, 2011, recruited participants, postmenopausal women, 62.4+/−4.9 years.

W. K. Kemmler, D. Lauber, K. Engelke, and J. Weineck, in “Effects of single- versus multiple-set resistance training on maximum strength and body composition in trained postmenopausal women,”* Journal of Strength and Conditioning Research*, vol. 18, no. 4, pp. 689–694, 2004, recruited participants 65–76 years of age, with undefined menopausal status.

L. R. Keytel, M. I. Lambert, J. Johnson, T. D. Noakes, and E. V. Lambert, in “Free living energy expenditure in post menopausal women before and after exercise training,”* International Journal of Sport Nutrition and Exercise Metabolism*, vol. 11, no. 2, pp. 226–237, 2001, recruited “postmenopausal” women, 58.7 ± 7 years of age, with no details on menopausal status.

L. H. Kuller, A. M. Kriska, and L. S. Kinzel et al., in “The clinical trial of Women On the Move through Activity and Nutrition (WOMAN) study,”* Contemporary Clinical Trials*, vol. 28, no. 4, pp. 370–381, 2007, recruited participants, women “postmenopausal,” 50–62 years of age with menopausal status being undefined.

L. H. Kuller, L. S. Kinzel, and K. K. Pettee et al., in “Lifestyle intervention and coronary heart disease risk factor changes over 18 months in postmenopausal women: The Women On the Move through Activity and Nutrition (WOMAN Study) clinical trial,”* Journal of Women's Health*, vol. 15, no. 8, pp. 962–974, 2006, recruited participants, women “postmenopausal,” 50–62 years of age with menopausal status being undefined.

A. Lapointe, S. J. Weisnagel, and V. Provencher, in “Comparison of a dietary intervention promoting high intakes of fruits and vegetables with a low-fat approach: long-term effects on dietary intakes, eating behaviours and body weight in postmenopausal women,”* British Journal of Nutrition*, vol. 104, no. 7, pp. 1080–1090, 2010, recruited participants, women “postmenopausal” (with absence of menses for at least one year and FSH levels), 45–68 years of age.

A. Lapointe, V. Provencher, and S. Weisnagel, in “Dietary intervention promoting high intakes of fruits and vegetables: short-term effects on eating behaviors in overweight-obese postmenopausal women,”* Eating Behaviors*, vol. 11, no. 4, pp. 305–308, 2010, recruited participants, women “postmenopausal” (with absence of menses for at least one year and FSH levels), 45–68 years of age.

S. G. Lee, I. G. Kim, and H. S. Yun et al., in “The effect of walking program on blood profile in obese middle-aged women. American Association of Cardiovascular and Pulmonary Rehabilitation 25th Annual Meeting 2010 Oct 6–Oct 9; Milwaukee, WI United States,”* Journal of Cardiopulmonary Rehabilitation and Prevention*, vol. 30, no. 4, pp. 275, 2010, recruited participants, “postmenopausal women,” with no additional details on menopausal status.

J. A. Lee, J. W. Kim, and D. Y. Kim, in “Effects of yoga exercise on serum adiponectin and metabolic syndrome factors in obese postmenopausal women,”* Menopause*, vol. 19, no. 3, pp. 296–301, 2012, recruited participants, “postmenopausal women (with absence of menses for one year+), 54.5+/−2.75 years and more than 36% body fat.”

Z. Liu, S. C. Ho, Y. Chen, and Y. P. Ho, in “A mild favorable effect of soy protein with isoflavones on body composition-a 6-month double-blind randomized placebo-controlled trial among Chinese postmenopausal women,”* International Journal of Obesity*, vol. 34, no. 2, pp. 309–318, 2010, recruited participants, postmenopausal women (greater than one year without menses), 48–70 years of age.

P. Llaneza, C. Gonzalez, and J. Fernandez-Inarrea J et al., in “Soy isoflavones, Mediterranean diet, and physical exercise in postmenopausal women with insulin resistance,”* Menopause*, vol. 17, no. 2, pp. 372–378, 2010, recruited participants: postmenopausal women (greater than or equal to one year without menses), 50–64 years of age.

P. J. Manns, D. P. Williams, C. M. Snow, and R. C. Wander, in “Physical activity, body fat, and serum C-reactive protein in postmenopausal women with and without hormone replacement,”* American Journal of Human Biology*, vol. 15, no. 1, pp. 91–100, 2003, recruited participants, postmenopausal women (with no menses in the past year), 50–73 years of age.

M. Moreira, V. Almeida, A. Araújo, M. Monteiro, J. Leitão, and F. Pitanga, in “The relationship between physical activity and body composition in two random groups of postmenopausal women. 8th European Congress on Menopause 2009 May 16–May 20; London, United Kingdom,”* Maturitas*, vol. 63, pp. S130, 2009, reported no details on menopausal status, “overweight postmenopausal women.”

M. Moreira, J. Brant, B. Ogando, R. Gabriel, and M. Monteiro, in “A randomized study about the effect of a 12-month exercise program in the body composition of postmenopausal women. Influence of some characteristics of menopause,”* Climacteric*, vol. 14, pp. 160, 2011, reported no details on menopausal status, “overweight postmenopausal women.”

H. S. Park and K. U. Lee, in “Postmenopausal women lose less visceral adipose tissue during a weight reduction program,”* Menopause*, vol. 10, no. 3, pp. 222–227, 2003, recruited “postmenopausal” women (with no menses for equal to or greater than one year), 57.6+/−7.2 years of age.

G. Schaberg-Lorei, J. E. Ballard, B. C. McKeown, and S. A. Zinkgraf, in “Body composition alterations consequent to an exercise program for pre and postmenopausal women,”* Journal of Sports Medicine and Physical Fitness*, vol. 30, no. 4, pp. 426–433, 1990, recruited women matching for age (range 35–70 years of age) (i.e., older group versus younger group), with no details on menopausal status.

M. Sénéchal, D. R. Bouchard, L. Soucy, I. J. Dionne, and M. Brochu, in “The impact of resistance training, with or without caloric restriction on body composition and the metabolic profile in obese postmenopausal women. 11th International Congress on Obesity 2010 Jul 11–Jul 15; Stockholm, Sweden,”* Obesity Reviews*, vol. 11, no. suppl. 1, pp. 43-44, 2010, recruited participants defined as “postmenopausal,” with no additional details.

M. Senechal, D. R. Bouchard, I. J. Dionne, and M. Brochu, in “The effects of lifestyle interventions in dynapenic-obese postmenopausal women,”* Menopause*, vol. 19, no. 9, pp. 1015–1021, 2012, recruited participants, women, 62.6+/−4.1 years of age, with FSH levels measured to determine postmenopausal status.

M. Skouroliakou, I. Giannopoulou, C. Kostara, K. Koutri, M. G. Stathopoulou, and C. Kakavelaki, in “Effects of a nutritional intervention in obese postmenopausal women on atypical antipsychotics,”* Maturitas*, vol. 67, no. 2, pp. 166–170, 2010, recruited participants, obese, postmenopausal women taking atypical antipsychotics, with no details on menopausal status.

O. L. Svendsen, C. Hassager, and C. Christiansen, in “The response to treatment of overweight in postmenopausal women is not related to fat distribution,”* International Journal of Obesity*, vol. 19, no. 7, pp. 496–502, 1995, recruited participants, overweight, postmenopausal women, 49–58 years of age.

A. Zarneshan and K. Salehzadeh, in “Effects of combined selective aerobic moderate intensity exercises and soya intake on 17ß-estradiol (biomarker of breast cancer) and obesity of obese postmenopausal women,”* International Journal of Biosciences*, vol. 2, no. 11, pp. 81–89, 2012, recruited participants, “menopausal” women, age of 49.6+/1.8 years, age in years 60.3+/−5.3 years, no reporting by menopausal age or defining menopausal age.

#### A.2. Mixed Pre-, Peri-, and/or Postmenopausal: Not Reported Separately (*n* = 28)

Consider the following references.

M. J. Caballero and M. Maynar, “Effects of physical exercise on sex hormone binding globulin, high density lipoprotein cholesterol, total cholesterol and triglycerides in postmenopausal women,”* Endocrine research*, vol. 18, no. 4, pp. 261–279, 1992.

H. Chihara, R. Kawase, Y. Otsubo, Y. Hiraizumi, and T. Takeshita, “Effect of insulin resistance improvement due to lifestyle intervention on overweight perimenopausal Japanese women: A preliminary study,”* Journal of Nippon Medical School*, vol. 75, no. 1, pp. 15–22, 2008.

A. F. G. Cicero, A. Dormi, S. D'Addato, A. V. Gaddi, and C. Borghi, “Long-term effect of a dietary education program on postmenopausal cardiovascular risk and metabolic syndrome: The brisighella heart study,”* Journal of Women's Health*, vol. 19, no. 1, pp. 133–137, 2010.

L. C. Dalleck, B. A. Allen, B. A. Hanson, E. C. Borresen, M. E. Erickson, and S. L. Lap, “Dose-response relationship between moderate-intensity exercise duration and coronary heart disease risk factors in postmenopausal women,”* Journal of Women's Health*, vol. 18, no. 1, pp. 105–113, 2009.

P. Deibert, D. Konig, M. Z. Vitolins, et al., “Effect of a weight loss intervention on anthropometric measures and metabolic risk factors in pre- versus postmenopausal women,”* Nutrition Journal*, vol. 6, pp. 31, 2007.

G. Fisher, G. R. Hunter, and B. A. Gower, “Aerobic exercise training conserves insulin sensitivity for 1 year following weight loss in overweight women,”* Journal of Applied Physiology*, vol. 112, no. 4, pp. 688–693, 2012.

M. Friedrich, “Effects of diet modification and the resultant body weight loss on body composition in obese menopausal women,”* Polish Journal of Food and Nutrition Sciences*, vol. 57, no. 4, pp. 503–508, 2007.

A. R. Josse, S. A. Atkinson, M. A. Tarnopolsky, and S. M. Phillips, “Increased consumption of dairy foods and protein during diet- and exercise-induced weight loss promotes fat mass loss and lean mass gain in overweight and obese premenopausal women,”* Journal of Nutrition*, vol. 141, no. 9, pp.1626–1634, 2011.

M. Kristensen, S. Toubro, M. G. Jensen, et al., “Whole grain compared with refined wheat decreases the percentage of body fat following a 12-week, energy-restricted dietary intervention in postmenopausal women,”* Journal of Nutrition*, vol. 142, no. 4, pp. 710–716, 2012.

A. J. Littman, M. V. Vitiello, K. Foster-Schubert, et al., “Sleep, ghrelin, leptin and changes in body weight during a 1-year moderate-intensity physical activity intervention,”* International Journal of Obesity*, vol. 31, no. 3, pp. 466–475, 2007.

S. Karacan, “Effects of long-term aerobic exercise on physical fitness and postmenopausal symptoms with menopausal rating scale,”* Science and Sports*, vol. 25, no. 1, pp. 35–46, 2010.

N. A. Lynch, B. J. Nicklas, D. M. Berman, K. E. Dennis, and A. P. Goldberg, “Reductions in visceral fat during weight loss and walking are associated with improvements in VO_2_ max,” Journal of applied physiology 2001; 90(1): 99–104.

N. Maesta, E. A. P. Nahas, J. Nahas-Neto, et al., “Effects of soy protein and resistance exercise on body composition and blood lipids in postmenopausal women,”* Maturitas*, vol. 56, no. 4, pp. 350–358, 2007.

A. K. Mahon, M. G. Flynn, L. K. Stewart, et al., “Protein intake during energy restriction: effects on body composition and markers of metabolic and cardiovascular health in postmenopausal women,”* Journal of the American College of Nutrition*, vol. 26, no. 2, pp. 182–189, 2007.

T. Nestares, L. F. Mde, J. Diaz-Castro, M. S. Campos, M. López-Frías, “Evaluating the effectiveness of a weight-loss program for perimenopausal women,”* International Journal for Vitamin and Nutrition Research*, vol. 79, no. 4, pp. 212–217, 2009.

B. J. Nicklas, X. W. Wang, T. J. You, et al., “Effect of exercise intensity on abdominal fat loss during calorie restriction in overweight and obese postmenopausal women: A randomized, controlled trial,”* American Journal of Clinical Nutrition*, vol. 89. No. 4, pp. 1043–1052, 2009.

F. L. Orsatti, E. A. Nahas, C. L. Orsatti, et al., “Muscle mass gain after resistance training is inversely correlated with trunk adiposity gain in postmenopausal women,”* Journal of Strength and Conditioning Research*, vol. 26, no. 8, pp. 2130–2139, 2012.

M. D. Phillips, R. M. Patrizi, D. J. Cheek, J. S. Wooten, J. J. Barbee, and J. B. Mitchell, “Resistance training reduces subclinical inflammation in obese, postmenopausal women,”* Medicine and Science in Sports and Exercise*, vol. 44, no. 11, pp. 2099–2110, 2012.

M. C. Poyatos and M. V. Vaquero, “Training in a shallow pool: Its effect on upper extremity strength and total body weight in postmenopausal women,”* International SportMed Journal*, vol. 12, no. 1, pp. 17–29, 2011.

A. E. Ready, B. Naimark, J. Ducas, et al., “Influence of walking volume on health benefits in women post-menopause,”* Medicine and Science in Sports and Exercise*, vol. 28, no. 9, pp. 1097–1105, 1996.

M. Roussel, S. Garnier, S. Lemoine, et al., “Influence of a walking program on the metabolic risk profile of obese postmenopausal women,”* Menopause*, vol. 16, no. 3, pp. 566–575, 2009.

S. A. Shapses, S. Heshka, and S. B. Heymsfield, “Effect of calcium supplementation on weight and fat loss in women,”* Journal of Clinical Endocrinology and Metabolism*, vol. 89, no. 2, pp. 632–637, 2004.

J. Shlisky, C. Durward, M. Zack, J. Campbell, S. Jonnalagadda, and S. Nickols-Richardson, “Including non-fat dairy in an energy-restricted (ER), moderate-protein diet plan: Two-week changes in body weight and composition measurements in premenopausal women with overweight and obesity,”* FASEB Journal*, vol. 25, pp. 593.3, 2011.

P. J. Teixeira, S. B. Going, L. B. Houtkooper, et al., “Resistance training in postmenopausal women with and without hormone therapy,”* Medicine and Science in Sports and Exercise*, vol. 35, no. 4, pp. 555–562, 2003.

S. P. Tokmakidis, C. E. Zois, K. A. Volaklis, K. Kotsa, and A. M. Touvra, “The effects of a combined strength and aerobic exercise program on glucose control and insulin action in women with type 2 diabetes,”* European Journal of Applied Physiology*, vol. 92, pp. 437–442, 2004.

G. Turner-McGrievy, N. D. Barnard, and A. R. Scialli, “A two-year randomized weight loss trial comparing a vegan diet to a more moderate low-fat diet,”* Obesity*, vol. 15, no. 9, pp. 2276–2281, 2007.

M. J. Velthuis, A. J. Schuit, P. H. M. Peeters, and E. M. Monninkhof, “Exercise program affects body composition but not weight in postmenopausal women,”* Menopause*, vol. 16, no. 4, pp. 777–784, 2009.

K. Z. Walker, K. O'Dea, and G. C. Nicholson, “Dietary composition affects regional body fat distribution and levels of dehydroepiandrosterone sulphate (DHEAS) in post-menopausal women with Type 2 diabetes,”* European Journal of Clinical Nutrition*, vol. 53, no. 9, pp. 700–705, 1999.

#### A.3. Identified as Premenopausal Women (e.g., No Menstrual Irregularities) or More Than 6 Years Postmenopausal Women (*n* = 24)

Consider the following references.

T. M. Asikainen, S. Miilunpalo, P. Oja, M. Rinne, M. Pasanen, and I. Vuori, “Walking trials in postmenopausal women: effect of one versus two daily bouts on aerobic fitness,”* Scandinavian Journal of Medicine and Science in Sports*, vol. 12, no. 2, pp.99–105, 2002.

M. Brochu, M. F. Malita, V. Messier, et al., “Resistance training does not contribute to improving the metabolic profile after a 6-month weight loss program in overweight and obese postmenopausal women,”* Journal of Clinical Endocrinology and Metabolism*, vol. 94, no. 9, pp. 3226–3233, 2009.

L. B. Bunyard, K. E. Dennis, and B. J. Nicklas, “Dietary intake and changes in lipoprotein lipids in obese, postmenopausal women placed on an American Heart Association Step 1 diet,”* Journal of the American Dietetic Association*, vol. 102, no. 1, pp. 52–57, 2002.

R. A. Carels, L. A. Darby, H. M. Cacciapaglia, and O. M. Douglass, “Reducing cardiovascular risk factors in postmenopausal women through a lifestyle change intervention,”* Journal of Women's Health*, vol. 13, no. 4, pp. 412–427, 2004.

L. O. Carmignani, A. O. Pedro, L. H. Costa-Paiva, and A. M. Pinto-Neto, “The effect of dietary soy supplementation compared to estrogen and placebo on menopausal symptoms: A randomized controlled trial,”* Maturitas*, vol. 67, no. 3, pp. 262–269, 2010.

L. M. Chiechi, G. Secreto, A. Vimercati, et al., “The effects of a soy rich diet on serum lipids: The Menfis randomized trial,”* Maturitas*, vol. 41, no. 2, pp. 97–104, 2002.

Colado JC, Triplett NT, Tella V, Saucedo P, Abellán J. Effects of aquatic resistance training on health and fitness in postmenopausal women,”* European Journal of Applied Physiology*, vol. 106, no. 1, pp. 113–122, 2009.

Z. R. Cordero-MacIntyre, T. G. Lohman, J. Rosen, et al., “Weight loss is correlated with an improved lipoprotein profile in obese postmenopausal women,”* Journal of the American College of Nutrition*, vol. 19, no. 2, pp. 275–284, 2009.

R. R. Costa, C. L. Alberton, M. Tagliari, and L. F. Martins Kruel, “Effects of resistance training on the lipid profile in obese women,”* Journal of Sports Medicine and Physical Fitness*, vol. 51, no. 1, pp. 169–177, 2011.

K. E. Dennis, N. Tomoyasu, S. H. McCrone, A. P. Goldberg, L. Bunyard, and B. B. Qi, “Self-efficacy targeted treatments for weight loss in postmenopausal women,”* Scholarly inquiry for nursing practice*, vol. 15, no. 3, pp. 259–276, 2001.

K. K. Han, J. M Jr. Soares, A. A. Haidar, G. R. Lima, and E. C. Baracat, “Benefits of soy isoflavone therapeutic regimen on menopausal symptoms,”* Obstetrics and Gynecology*, vol. 99, no. 3, pp. 389–394, 2002.

V. Leblanc, V. Provencher, C. Begin, L. Corneau, A. Treblay, and S. Lemieux, “Impact of a Health-At-Every-Size intervention on changes in dietary intakes and eating patterns in premenopausal overweight women: Results of a randomized trial,”* Clinical Nutrition*, vol. 31, no. 4, pp. 481–488, 2011.

R. Monteiro, P. T. A. Riether, and R. C. Burini, “The effects of a mixed program of nutritional intervention and physical exercise on body composition and feeding habits of obese climacteric women./Efeito de um programa misto de intervenção nutricional e exercício físico sobre a composição corporal e os hábitos alimentares de mulheres obesas em climatério,”* Revista de Nutrição*, vol. 17, no. 4, pp. 479–489, 2004.

B. J. Nicklas, “Regional differences in basal and hormone-stimulated* in vitro* lipolysis and the effects of short term, low-intensity endurance exercise on lipolysis in obese, postmenopausal women[dissertation],”* University of Maryland*, Maryland, 1994.

B. J. Nicklas, E. M. Rogus, and A. P. Goldberg, “Exercise blunts declines in lipolysis and fat oxidation after dietary-induced weight loss in obese older women,”* American Journal of Physiology*, vol. 273, no. 1, pp. E149–E155, 1997.

M. T. Restrepo Calle, A. Monroy de Peña, J. P. Giraldo, and M. C. Velásquez Echeverri, “The effect of controlled physical activity on the body composition of postmenopausal sedentary women./Efecto de la actividad física controlada sobre la composición corporal de mujeres sedentarias posmenopáusicas. Revista Panamericana de Salud Pública/Pan,”* American Journal of Public Health*, vol. 14, no. 4, pp. 229–234, 2003.

E. Riesco, M. Aubertin-Leheudre, M. L. Maltais, M. Audet, and I. J. Dionne, “Synergic effect of phytoestrogens and exercise training on cardiovascular risk profile in exercise-responder postmenopausal women: A pilot study,”* Menopause*, vol. 17, no. 5, pp. 1035–1039, 2010.

I. Castan-Laurell, M. Vitkova, D. Daviaud, et al., “Effect of hypocaloric diet-induced weight loss in obese women on plasma apelin and adipose tissue expression of apelin and APJ,”* European Journal of Endocrinology*, vol. 158, no. 6, pp. 905–910, 2008.

P. Hanachi and S. Golkho, “Assessment of soy phytoestrogens and exercise on lipid profiles and menopause symptoms in menopausal women,”* Journal of Biological Sciences*, vol. 8, no. 4, pp. 789–793, 2008.

C. Lee and S. W. White, “Controlled trial of a minimal-intervention exercise program for middle-aged working women,”* Psychology and Health*, vol. 12, no. 3, pp. 361–374, 1997.

J. M. Lukaszuk, P. Luebbers, and B. A. Gordon, “Soy milk as effective as skim milk in promoting weight loss,”* Journal of the American Dietetic Association*, vol. 107, no. 10, pp. 1811–1814, 2007.

E. Riesco, S. Tessier, F. Perusse, et al., “Impact of walking on eating behaviors and quality of life of premenopausal and early postmenopausal obese women,”* Menopause*, vol. 17, no. 3, pp. 529–538, 2010.

J. S. Wooten, M. D. Phillips, and J. B. Mitchell, “Resistance Exercise and Lipoproteins in Postmenopausal Women,”* International Journal of Sports Medicine*, vol. 32, no. 1, pp. 7–13, 2011.

T. Y. Wu, H. I. Yeh, P. Chan, Y. F. Chiou, and J. C. Tsai, “The effects of simple eight-week regular exercise on cardiovascular disease risk factors in middle-aged women at risk in Taiwan,”* Acta Cardiologica Sinica*, vol. 23, no. 3, pp. 169–176, 2007.

#### A.4. Not an Intervention Design (*N* = 7)

##### A.4.1. Evidence on the Role of Exercise (*n* = 2)

Consider the following references.

W. M. Kohrt, “Menopause medicine: Exercise and weight gain,”* Geriatrics*, vol. 64, no. 6, pp. 28-29, 2009.

J. M. Lutter and K. Grumstrup, “Physical activity and weight in the menopausal years. The Melpomene/SELF magazine study,”* Melpomene Journal*, vol. 13, no. 1, pp. 17–23, 1994.

##### A.4.2. Role of Diet (*n* = 1)

Consider the following reference.

L. Hallberg and A. Svanborg, “Cholesterol, phospholipids, and triglycerides in plasma in 50-year-old women. Influence of menopause, body-weight, skinfold thickness, weight-gain, and diet in a random population sample,”* Acta Medica Scandinavica*, vol. 181, no. 2, pp. 185–194, 1967.

##### A.4.3. Other Issues Associated with Menopause (*n* = 4)

Consider the following references.

F. Aragao, C. Abrantes, R. Gabriel, and H. Moreira, “Effects of an exercise program on cardiorespiratory fitness and body composition in postmenopausal women,”* Climacteric*, vol. 14, pp. 104.

D. Bitner and R. F. Wild, “A clinical method to reduce waist circumference and menopausal symptoms in women aged 35–55,”* Menopause*, vol. 19, no. 12, pp. 1384, 2012.

D. L. Swift, N. M. Johannsen, C. Tudor-Locke, et al., “Exercise training and habitual physical activity: a randomized controlled trial,”* American Journal of Preventive Medicine*, vol. 43, no. 6, pp. 629–635, 2012.

G. M. Timmerman and A. Brown, “The effect of a mindful restaurant eating intervention on weight management in women,”* Journal of Nutrition Education* &* Behavior*, vol. 44, no. 1, pp. 22–28, 2012.

#### A.5. Wrong Outcomes (*n* = 16)

##### A.5.1. Bone Health (*n* = 3)

Consider the following references.

I. Bergström, C. Lombardo, and J. Brinck, “Physical training decreases waist circumference in postmenopausal borderline overweight women,”* Acta Obstetricia et Gynecologica Scandinavica*, vol. 88, no. 3, pp. 308–313, 2009.

Y. Manios, G. Moschonis, K. Koutsikas, et al., “Changes in body composition following a dietary and lifestyle intervention trial: The postmenopausal health study,”* Maturitas*, vol. 1, no. 58, pp. 65, 2009.

L. R. Simkin-Silverman, R. R. Wing, M. A. Boraz, E. N. Meilahn, and L. H. Kuller, “Maintenance of cardiovascular risk factor changes among middle-aged women in a lifestyle intervention trial,”* Womens Health*, vol. 4, no. 3, pp. 255–271, 1998.

##### A.5.2. Quality of Life (*n* = 1)

Consider the following reference.

S. Elavsky and E. McAuley. “Physical activity and mental health outcomes during menopause: A randomized controlled trial,”* Annals of Behavioral Medicine*, vol. 33, no. 2, pp. 132–142, 2007.

##### A.5.3. Other Outcomes Unrelated to Study (*n* = 12)

Consider the following references.

F. Arslan, E. Çakmakçi, H. Taskin, O. Çakmakçi, and C. G. Ismet, “Evaluation of the Effects of Pilates Mat Exercise Program on some Fitness Parameters and Weight Loss of Middle Aged Perimenopausal Sedentary Women./Orta YaSli PerImenopozal Sedanter Bayanlarda PIlates Mat EgzersIz Programinin KIlo Kaybi Ve FIzIksel Uygunluk ParametrelerI ÜzerIne EtkIlerInIn DeGerlendIrIlmesI,”* Journal of Physical Education* &* Sports Science/Beden Egitimi ve Spor Bilimleri Dergisi*, vol. 6, no. 1, pp. 24–33, 2012.

T. E. Brinkley, X. Wang, N. Kume, H. Mitsuoka, and B. J. Nicklas, “Caloric restriction, aerobic exercise training and soluble lectin-like oxidized LDL receptor-1 levels in overweight and obese post-menopausal women,”* International journal of obesity*, vol. 35, no. 6, pp. 793–799, 2011.

C. Y. Doyon, M. Brochu, V. Messier, et al., “Association between Abdominal Fat (DXA) and Its Subcomponents (CT Scan) before and after Weight Loss in Obese Postmenopausal Women: A MONET Study,” Journal of Obesity, vol. 2011, Article ID 239516, 6 pages, 2011. http://dx.doi.org/10.1155/2011/239516.

H. S. Lee, J. W. Lee, J. M. Kim, and N. S. Chang, “The effect of nutrition education and exercise program on body composition and dietary intakes, blood lipid and physical fitness in obese women (2)—Relationship between participation rates and effectiveness of obesity management program,”* Korean Journal of Nutrition*, vol. 43, no. 3, pp. 260–272, 2010.

L. E. Moeller, C. T. Peterson, K. B. Hanson et al., “Isoflavone-rich soy protein prevents loss of hip lean mass but does not prevent the shift in regional fat distribution in perimenopausal women,”* Menopause*, vol. 10, no. 4, pp. 322–331, 2003.

M. C. Mojtahedi, M. P. Thorpe, and D. C. Karampinos, “The effects of a higher protein intake during energy restriction on changes in body composition and physical function in older women,”* Journals of Gerontology. Series A, Biological Sciences and Medical Sciences*, vol. 66, no. 11, pp. 1218–1225, 2011.

M. H. Moreira, L. M. Vaz, J. S. Rocha, B. M. Ogando, and R. E. Gabriel, “Effects of exercise training on climacteric symptoms of postmenopausal women: A randomized study,”* Maturitas*, vol. 71, pp. S66, 2012.

N. S. Polis. Aerobic exercise: Effects on selected health variables in menopausal women [dissertation],”* Case Western Reserve University, Cleveland, OH*, 1989.

E. Riesco, S. Choquette, M. Audet, J. Lebon, D. Tessier, and I. J. Dionne, “Effect of exercise training combined with phytoestrogens on adipokines and C-reactive protein in postmenopausal women: a randomized trial,”* Metabolism, Clinical and Experimental*, vol. 61, no. 2, pp. 273–280, 2012.

J. Rocha, B. Ogando, R. Gabriel, M. Monteiro, and H. Moreira, “Effect of a 12-month exercise program on body composition of postmenopausal women: Effects of the nature of menopause,”* Menopause*, vol. 18, no. 12, pp. 1369, 2011.

J. S. Rocha, B. Ogando, M. S. Rocha, M. H. Moreira, R. Gabriel,R and V. Reis, “Impact of a 12-month exercise program and hormone therapy (HT) on the body composition of postmenopausal women,”* Menopause*, vol. 9, no. 12, pp. 1396, 2012.

S. W. Washburn, G. L. Burke, T. Morgan, and M. Anthony. “Effect of soy protein supplementation on serum lipoproteins, blood pressure, and menopausal symptoms in perimenopausal women,”* Menopause*, vol. 6, no. 1, pp. 7–14, 1999.

## References

[1] Statistics Canada http://www.statcan.gc.ca/daily-quotidien/100113/dq100113a-eng.htm.

[2] Statistics Canada http://www.statcan.gc.ca/tables-tableaux/sum-som/l01/cst01/health81b-eng.htm.

[3] World Health Organization http://www.who.int/gho/ncd/risk_factors/obesity_text/en/.

[4] Statistics Canada http://www.statcan.gc.ca/tables-tableaux/sum-som/l01/cst01/health77b-eng.htm.

[5] Statistics Canada http://www.statcan.gc.ca/tables-tableaux/sum-som/l01/cst01/health90a-eng.htm.

[6] Harlow SD, Gass M, Hall JE (2012). Executive summary of the stages of reproductive aging workshop +10: addressing the unfinished agenda of staging reproductive aging. *Fertility and Sterility*.

[7] Lovejoy JC, Champagne CM, De Jonge L, Xie H, Smith SR (2008). Increased visceral fat and decreased energy expenditure during the menopausal transition. *International Journal of Obesity*.

[8] Gingrich PM, Fogel CI (2003). Herbal therapy use by perimenopausal women. *Journal of Obstetric, Gynecologic, and Neonatal Nursing*.

[9] Whiteman MK, Staropoli CA, Langenberg PW, McCarter RJ, Kjerulff KH, Flaws JA (2003). Smoking, body mass, and hot flashes in midlife women. *Obstetrics and Gynecology*.

[10] Thurston RC, Sowers MR, Sternfeld B (2009). Gains in body fat and vasomotor symptom reporting over the menopausal transition: the study of women's health across the nation. *The American Journal of Epidemiology*.

[11] Lovejoy JC (2009). Weight gain in women at midlife: the influence of menopause. *Obesity Management*.

[12] Dennis KE (2007). Postmenopausal women and the health consequences of obesity. *Journal of Obstetric, Gynecologic, and Neonatal Nursing*.

[13] Schmitz KH, Hannan PJ, Stovitz SD, Bryan CJ, Warren M, Jensen MD (2007). Strength training and adiposity in premenopausal women: strong, Healthy, and Empowered study. *The American Journal of Clinical Nutrition*.

[14] Sternfeld B, Bhat AK, Wang H, Sharp T, Quesenberry CP (2005). Menopause, physical activity, and body composition/fat distribution in midlife women. *Medicine and Science in Sports and Exercise*.

[15] Agency for Healthcare Research Quality http://www.guideline.gov/content.aspx?id=33134&search=obesity.

[16] Lovejoy JC (1998). The influence of sex hormones on obesity across the female life span. *Journal of Women's Health*.

[17] Davis SR, Castelo-Branco C, Chedrani P (2012). Understanding weight gain at menopause. *Climacteric*.

[18] Simkin-Silverman LR, Wing RR, Boraz MA, Kuller LH (2003). Lifestyle intervention can prevent weight gain during menopause: results from a 5-year randomized clinical trial. *Annals of Behavioral Medicine*.

[19] Ogwumike OO, Arowojolu AO, Sanya AO (2011). Effects of a 12-week endurance program on adiposity and flexiblity of Nigerian perimenopausal and postmenopausal women. *Nigerian Journal of Physiological Sciences*.

[20] Hagner W, Hagner-Derengowska M, Wiacek M, Zubrzycki IZ (2009). Changes in level of VO2max, blood lipids, and waist circumference in the response to moderate endurance training as a function of ovarian aging. *Menopause*.

[21] Higgins JPT, Green S

[22] Moher D, Liberati A, Tetzlaff J, Altman DG, The Prisma Group (2009). Preferred reporting items for systematic reviews and meta-analysis: The PRISMA statement. *Open Medicine*.

[23] Grant M, Booth A (2009). A typology of reviews: an analysis of 14 review types and associated methodologies. *Health Information and Libraries Journal*.

[24] Stillwell SB, Fineout-Overholt E, Melnyk BM, Williamson KM (2010). Evidence-based practice, step by step: asking the clinical question: a key step in evidence-based practice. *The American Journal of Nursing*.

[25] Hartling L, Ospina M, Liang Y (2009). Risk of bias versus quality assessment of randomised controlled trials: cross sectional study. *British Medical Journal*.

[26] Critical Skills Appraisal Programme http://www.casp-uk.net/.

[27] Lau DCW, Douketis JD, Morrison KM, Hramik IM, Sharma EU (2007). Guidelines on the management and prevention of obesity in adults and children: Executive summary. *Canadian Medical Association Journal*.

[28] Vance VA, Hanning R, McCargar L (2007). Combined diet and exercise therapy for the treatment of obesity in adults. *Canadian Medical Association Journal*.

[29] Williams L, Germov J, Young A (2007). Preventing weight gain: a population cohort study of the nature and effectiveness of mid-age women's weight control practices. *International Journal of Obesity*.

[30] Abdulnour J, Doucet É, Brochu M (2012). The effect of the menopausal transition on body composition and cardiometabolic risk factors: a Montreal-Ottawa new emerging team group study. *Menopause*.

[31] Soules MR, Sherman S, Parrott E (2001). Executive summary: Stages of Reproductive Aging Workshop (STRAW) Park City, Utah, July, 2001. *Menopause*.

[32] Katzmarzyk PT (2007). Epidemiology of Obesity. *CMAJ*.

[33] Williams L, Germov J, Young A (2011). The effect of social class on mid-age women's weight control practices and weight gain. *Appetite*.

